# Older women’s experiences of companion animal death: impacts on well-being and aging-in-place

**DOI:** 10.1186/s12877-021-02410-8

**Published:** 2021-08-23

**Authors:** Donna M Wilson, Leah Underwood, Eloise Carr, Douglas P Gross, Morgan Kane, Maxi Miciak, Jean E Wallace, Cary A Brown

**Affiliations:** 1 Faculty of Nursing, University of Alberta, Edmonton, Canada; 2 Faculty of Nursing, University of Calgary, Calgary, Canada; 3 Department of Physical Therapy, Faculty of Rehabilitation Medicine, University of Alberta, Edmonton, Canada; 4 Department of Occupational Therapy, Faculty of Rehabilitation Medicine, University of Alberta, 2–64 Corbett Hall, Alberta T6G2G4 Edmonton, Canada; 5 Faculty of Arts, University of Calgary, Alberta Calgary, Canada

**Keywords:** Companion animal, Death, Grief, Older adult, Women, Social support, Aging in place, Well-being

## Abstract

**Background:**

Companion animal death is a common source of grief, although the extent and context of that grief is poorly understood, especially in older adulthood. The aim of this multiple-methods study was to develop a greater understanding of the impact of companion animal death on older women living alone in the community, as older women are a distinct at-risk group, and the supports that should be available to help these individuals with their grief.

**Methods:**

Participants were recruited from across Alberta, a Canadian province, through seniors’ organizations, pet rescue groups, and social media groups of interest to older women. After completing a pre-interview online questionnaire to gain demographic information and standardized pet attachment and grief measures data, participants were interviewed through the Zoom ® computer program or over the telephone. An interpretive description methodology framed the interviews, with Braun and Clarke’s 6-phase analytic method used for thematic analysis of interview data.

**Results:**

In 2020, twelve participants completed the pre-interview questionnaires and nine went on to provide interview data for analysis. All were older adult (age 55+) women, living alone in the community, who had experienced the death of a companion animal in 2019. On the standardized measures, participants scored highly on attachment and loss, but low on guilt and anger. The interview data revealed three themes: catastrophic grief and multiple major losses over the death of their companion animal, immediate steps taken for recovery, and longer-term grief and loss recovery.

**Conclusions:**

The findings highlight the importance of acknowledging and addressing companion animal grief to ensure the ongoing well-being and thus the sustained successful aging-in-place of older adult women in the community.

## Background

One of the most common adverse events for people in older age groups is the death of a loved one [1]. Not only do these deaths greatly increase their risk of immediate and ongoing social isolation, but they are also a major factor in the initiation of new or exacerbated illnesses arising from persistent anxiety and depression, such that premature mortality can result [1]. People of all ages may experience severe and unrelenting and thus highly impactful grief [1]. However, the normal emotional, mental and other impacts of bereavement grief are compounded for older people as they typically have multiple age-related challenges such as reduced income, lifestyle and housing changes with retirement, reductions in their social support networks, and declining health and physical strength [2]. The likelihood of older women in particular to have fewer financial resources, and to outlive friends and family, exacerbates the impact of these age-related challenges for them, while contributing greatly to a reduced likelihood of their successful aging-in-place [3].

A common but often under-recognized source of major grief is companion animal (CA) death. Companion animals, often described as pets, typically reside with humans who describe their relationship with the animal to be emotionally meaningful, most frequently because of the social companionship that this animal provides [4]. The scientific scrutiny of this caring human-animal cohabitation phenomenon is only just emerging [4–12]. Consequently, CA grief is poorly understood by those responsible for providing social support services or healthcare interventions and also by friends and family members [7–13]. This knowledge gap is highly concerning given that CAs appear to be an international reality. For example, it has been reported that CAs are part of 67 % of American households; moreover, there are 1.4 CAs for every Australian [9]. Unfortunately, most CAs have a shorter, if not much shorter, lifespan than their owners, with grief a possibility if not eventuality when these CAs die. While some developments in educating veterinary professionals about CA grief have occurred [14–16], there has been little attention paid to establishing CA grief training and resources for social service, healthcare, and pastoral care professionals involved in providing services for the immediate or ongoing health and emotional needs of older adults [4, 5, 16]. Appropriate services for older people are particularly needed for grief of all kinds to reduce the risk of institutionalization through enabling successful aging-in-place.

The service gap for older adults grieving a companion animal death is surprising, as the benefits of the CA/human relationship for optimal aging is clearly demonstrated in the research literature. Companion animal relationships with older adults have been associated with increased physical activity, reduced pain, better sleep, improved feelings of safety and security, emotional support, sense of purpose, greater perceptions of acceptance, and increased opportunities for the formation of social relationships; all important factors contributing to successful aging in the community [2, 17–21]. Losing these benefits when a CA dies can be of significant negative impact. Addressing this research gap, we undertook a study to better understand the extent and context of grief related to the death of a CA for older women. We also explored the supports and interventions they utilized in addressing their grief. While recognizing that people of all genders can experience significant bereavement grief we chose to focus this study on women. As indicated previously, they have unique factors that impact aging-in-place such as an increased likelihood of having fewer financial resources, living alone in old age, and outliving friends and family who have provided social and emotional support in the past [3].

## Methods

This was a multiple methods study, with a pre-interview standardized measures component followed by qualitative interviews. Due to the scarcity of existing knowledge about older women’s CA grief and recovery, we thought it was important to gain insight from women themselves through giving voice to their own experiences. This personal experiential focus is important for our developing a strong conceptual foundation for grief recovery, particularly in relation to maintaining successful aging-in-place. Interpretive description (ID), a qualitative methodological approach that addresses problems requiring practical insight, was chosen to inform the interview data collection and subsequent analysis for this study [22]. The ID qualitative approach seeks to gain insight into participant lived experiences and knowledge as required for an in-depth understanding of a complex phenomenon (in this case CA grief and its impact on the wellbeing of older women living alone in the community) [22]. The inherent flexibility of the approach allows for a deep examination of complex questions, particularly those requiring practical strategies to address [22].

Braun and Clarke’s 6-step analytic method was also selected because it is an iterative approach that allows for theory-informed (as opposed to theory-driven) data examination; a method that is widely used in emerging areas of study [23], such as CA bereavement. Braun and Clarke’s analytic method is one of the most widely cited approaches to qualitative data analysis, and its clear stepwise method lends itself to large data sets and also to team analysis, such as was carried out in this study [23]. This analysis approach was utilized with the aim of informing decision-makers and practitioners about the management of CA grief and for theory development to build targeted CA bereavement interventions.

To thoroughly characterize and describe the participants, we first collected descriptive demographic data and administering two standardized instruments, the Pet Attachment and Life-Impact Questionnaire (PALS) [24] and the Pet Bereavement Questionnaire (PBQ) [25]. The PALS is a psychometrically tested tool assessing owner attachment, and perceived negative and positive aspects of the CA relationship to the pet owner’s life. The PALS has 39 questions asking each participant’s level of agreement with statements on a 5-point scale (“1 = not at all” to “5 = very much”) about their pet in regards to four factors: “Love,” “Regulation,” “Personal Growth,” and “Negative Impact”. Higher scores indicate greater endorsement of impact. The PBQ includes 16 questions, each using a 4-point Likert scale to answer (0 = Disagree strongly, 1 = Disagree, 2 = Agree, 3 = Agree Strongly) composed of three factors: “Grief”, “Anger”, and “Guilt”. Higher scores signifying higher degrees of bereavement distress. Neither tool has normative data. The demographic, consent, and questionnaire data were collected online before the interviews of those who agreed to this second data collection component were conducted.

Research ethics approval was obtained in advance from the University of Alberta Health Research Ethics Board (Pro00101778) and also the University of Calgary Conjoint Health Research Ethics Board (REB20-1094). Research rigour was strengthened through having a team member who is an experienced qualitative data analyst complete the qualitative data analysis, with this analysis reviewed by another team member and confirmed as correct. Rigour was also strengthened by employing and carefully training and monitoring the work of one consistent interviewer who was not part of the study conceptualization. Moreover, to reduce the risk of confirmation bias, the preliminary interview data coding was completed by a research assistant who was not part of the study conceptualization. After this preliminary coding, the analysis was refined by other team members through repeated deep readings of the transcripts and then team discussion.

To strengthen theoretical concept development and maximize sample variation, a province-wide study across the Canadian province of Alberta was conducted. Recruitment occurred in 2020 through the province’s seniors’ organizations, community stakeholders (e.g. Alberta Association of Seniors Centres, Alberta Veterinary Medical Association and Veterinary Technicians’ Association), pet rescue groups, and social media platforms of Alberta-based groups where the population of interest were likely to be members (e.g. gardening and women’s hiking groups).

The targeted participants were older (age 55+) women living alone in the community, who had experienced the loss of a CA in 2019. Interested volunteers contacted the primary investigator (PI) by email. After screening for eligibility, they were forwarded the URL to complete the online consent and pre-interview demographic data form about themselves and their CA, and the PALS and PBQ. After those forms and questionnaires were completed, the research assistant (RA) contacted each participant who volunteered for an interview to schedule and carry out the interview.

Verbal consent was collected by the RA before the start of each arranged interview. An interview schedule of predetermined questions was consistently used with minor modifications as the study progressed across all interviews, and each interview lasted 30–60 min. All interviews took place in the months of July through November 2020, and all were recorded using the ZOOM ® computer platform or by an audiotape when telephone interviews occurred. Interviews focused on identifying the impact of CA death on the participants, what participants believed was helpful for recovering from their grief, any informal or formal bereavement interventions utilized, and what supports they believe could have helped them. Interview transcripts, generated automatically by the Zoom platform following recording, were downloaded and anonymized by the principal investigator (PI). The RA who carried out the interview then screened the transcript for completeness and accuracy within two days of each interview. Interviews carried out by phone were taped and transcribed by the RA.

The research team, who had been monitoring the interviewing and gathered data, determined data saturation occurred at nine participants when they noted increasingly repetitive data across participants, and gained no additional new understandings of the data. One of the primary investigators then independently coded the data and drafted themes and sub-themes, before discussing these findings with other members of the research team who reviewed, revised, and then affirmed the final themes and sub-themes. After this, in keeping with Braun and Clarke’s 6-phase data analysis method [20], this research report was drafted in which the themes and sub-themes were again reviewed and confirmed by all team members. The selection of quotes that best illustrate the concepts and relationships between themes and sub-themes was also confirmed at this point.

## Results

Twelve women met the criteria and participated in this study. They ranged in age from 57 to 85 years, with 65 the average age. Each of the participants had experienced a CA loss (10 dogs, 2 cats) in 2019 (all pre-COVID) after living with their CA for 13 or more years. At the time of CA death, participants were living alone in the community in either a house, apartment, or condominium. Four participants were living in a town with a population of 1,000 to 50,000, 1 participant lived in a small city with a population between 50,001 and 100,000, and 7 participants lived in a large city with a population of 200,000 or more.

The 12 participants who completed the online questionnaires had a high mean score on the PALS [21] life-impact factor “Love” (4.7 of a possible 5), followed less notably by “Personal growth” (3.6) and “Self-regulation” (3.41). Their mean score on the life-impact factor “Negative” was quite low at 1.2. On the PBQ [22] participants’ mean scores were highest on the bereavement-factor “Grief” (14.75; range 0–21), followed by much lower scores on the bereavement-factors “Anger” (2.75; range 0–15) and “Guilt” (2.25; range 0–12).

Eight participants had more than one CA at the time of the reported CA death, while for four participants, this had been their only CA. Companion animal death primarily occurred through euthanasia, either at the participant’s home or at a veterinary office, with a veterinarian present. For some participants, euthanasia of their CA was required quickly and unexpectedly, as they were unaware of an underlying illness. One participant was not directly involved in making the decision to euthanize her CA as she were away from home and were only told of her CA’s death through euthanasia after it had been arranged by a friend who was looking after her CA in her absence.

Three participants declined the qualitative interview component of the study; one stated a lack of time and two others stating it was too stressful given other personal challenges with the COVID-19 epidemic. Nine participants were subsequently interviewed about their experiences of CA loss. From the interview data, three primary themes were identified, with additional sub-themes occurring within each primary theme. Each theme has direct quotes chosen from participant interviews to demonstrate and validate the relevance of each.

### Catastrophic grief and multiple major losses associated with the death of their CA

#### Intense and enduring grief

All participants reported significant grief after the loss of their CA, with most participants categorizing their grief at 10/10. This high-level grief continued for weeks, if not months, with some participants rating their grief during the interview as 6/10, although it had been a year or more since their CA death. This level of reported grief aligns with the participants’ overall high score on the PBQ bereavement-factor “Grief” (14.75).

P5. *“It left a huge void. It was really bad…You’re very, very sad, you know, for 2, 3, 4 or 5 months.”*

P6. *“I cried a lot. I cried, an awful lot and I cried myself to sleep that night.”*

P8. *“This is something that takes over your life and takes that control away from you because you cannot…you can’t find a way to mitigate it. And it’s just there. And you have to deal with it. So yeah, it is painful and it does bring you down.”*

#### Loss of social activities associated with the CA

Many immediate social losses resulted from the CA death. For example, participants reported meeting other people through their CA, with that possibility now gone. Their CA also created opportunities for discussion over pet-related and then other matters. In addition, many participants got to know their community and neighbors through walks with their CA or through other pet-related activities.

P2. *“When we go out for walks, I’d sort of just let him take the lead whichever way you want to turn right or turn left or whatever. That would be fine. And that’s how we got to know the neighborhood, but we also got to meet a lot of people that way as well. So if he hadn’t been with me, I wouldn’t have met those people. I wouldn’t have learned the area.”*

P8. *“I actually got into dog training because of him. I met so many people because of him. I got into rescue because of him. He really was the dog that changed everything going forward. Oh yeah, he was a big part of my life.”*

P5. *“I still went walking because I like being outside, but you don’t connect with people as easy…With dogs, it’s a more communal thing. You walk somewhere, somebody else has a dog and you sort of, oh, you know, let’s walk together and go for an hour and it can be so random and doesn’t always happen all the time, but when it does, it’s nice, you know, we walk, then by the end you have their names.”*

#### Loss of personal relationship with, and daily support from, their CA

Participants reported that their CA had been a constant or consistent source of in-house company, and was a source of much laughter and entertainment. Participants felt their CA knew them, were attuned to their feelings and thoughts, responded to them, were always glad to see them, and offered unconditional love and acceptance. In most instances, the pet lived many years with the participant (mean duration of 14.8 years) and was like a child or family member to them. Many participants had raised their CA from shortly after the CA’s birth.

P3. *“I have no children and so he was kind of it. Yeah. He was the only dog that I’ve ever had from a baby. So I think that bond was created…I can’t consider him my sole reason for existing but I think with a lot of older people, if you’re alone, it makes a massive difference to have…you’re not coming home to dead air.”*

P5. *“With a dog you always have company. They’re always true. You know, if you know dogs, your dog looks at you. When they look at us and they know what’s going on, you know, they have a good radar and they have a good sense of humor…It was a tight bond and we have been just the two of us for years…And if I would think of going for a walk she would walk to the door. It was uncanny, like she really knew what I was up to.”*

P12. *“[H]e got me walking. We walked a lot, especially when he was younger…He was there for me during divorce and some tough times. And, you know, just being there.”*

### Immediate steps for recovery

#### First steps in initial death-associated actions

Participants typically ensured and approved timely and humane euthanasia of their CA. Conversations with the veterinarian, veterinary staff, and/or close family members occurred before and also after the death to confirm that euthanasia was the right decision to end the suffering of their animal. Participants felt their actions were based on compassion and concern for their CA, and they consoled themselves that day, and in the following days, as they remembered and approved their decision.

P6. *“It was just really, really hard and I wish, I didn’t know what to do. My son…I was talking to him and telling him that I just wasn’t sure what I should do about it. And he says, well take her to the vet and see what the vet says…I made an appointment… within a couple of hours of taking her to the vet and she couldn’t even walk and she’s having a really hard time breathing. So there was just like, no question about it…It was good for me because I didn’t really have time to make a decision, it was obvious what had to happen.”*

P10. *“She was sick and suffering and there was a relief that she wasn’t suffering anymore. That cuts into the real grief, is to know that she is finally not suffering.”*

P12. *“I talked to the vet, and a friend went with me to the vet that day. It was obvious that it was coming. My friend provided support, and the vet was very considerate and the vet even offered to drive me home if needed.”*

#### Hunkering down

Participants recognized and accepted their initial major grief, most choosing to do little but stay home and grieve. They were careful in who they told about the death of their CA so as to gain helpful immediate support from a select few friends or family members and to avoid people they believed would be unhelpful or dismissive of their grief. Participants purposely did not notify or tell select people, instead seeking and gaining support from a few key persons who they were confident would understand. Some participants even gave directions or expectations on social media that they wanted to be left alone, as they were grieving.

P3. *“That day, my good friend came with me and then she and I came back here, and we hung out for an hour. Then she went home and I didn’t have to go to work for about five days and I needed every one of those days. Then I just sort of had to let people know. I think I posted something on Facebook just so I didn’t have to phone people or for people to not know and just call and say, ‘How’s it going’ sort of thing. So that’s, yeah, that’s kind of what I did.”*

P8. *“I guess people were more reaching out to me than I was reaching out to them. Okay, so there was like I say, a lot of support and I appreciate that. But I was not inclined to reach out to other people. For one thing, even after a year, I can get all weepy about it. So talking about when it was fresh back then…. I couldn’t do it and… I even had talked to my manager about when I come back because they gave me a day off of work or two, but they called it family time they were very nice about it so I didn’t have to take time off, like when I asked her to kind of brief everybody not to bring it up and not to say anything to me about it. I just wanted to come to work and be normal. And not have that invade that world as well.”*

P8. *“You always feel angry at the vet and I know that that’s misplaced and you always think that they could have done something…That’s a really big part of the grieving process that you have by that and know that you made the right decision. And that’s where you need other people to help because you could just really start festering that feeling for a long time. If you don’t have somebody that just says, yeah, you made the right decision and just kind of validate that for you.”*

### Longer-term grief and loss recovery

#### Starting to move on

Some participants reported finding ways to manage their grief over time. Others did not report specific activities targeted to grief-management but rather talked about ways that they began to overcome the losses in their social and physical activities that happened when their CA died. None reported seeking or obtaining help through formal bereavement support services or religious/spiritual support from organized religions. Some participants began to go out and be active again in pet-related services or pet rescue societies. Others chose non-CA related activities in which to become active again. Participants resumed walks and some other social activities alone or with a remaining pet, and disclosed the death of their CA to a few more people. Personal and shared memories of their deceased CA were often sought from others, some participants received mementoes of the CA from friends, and participants reported keeping memorials of their deceased CA such as paw prints, pictures, and/or cremated remains. Some participants reported they were still managing considerable ongoing grief as they found the bond with their CA was deep and irreplaceable.

P2. *“One thing I did was I joined, I volunteer with (pet rescue organization). Got the information about that program and as a volunteer walker, I thought, well at least I’ll have contact with a dog, it’ll keep me out getting exercise and everything.”*

P7. *“I was taking his ashes to a spot that he always loved to walk and it was a spot that was special to me. There’s crocuses there every spring, it’s just kind of a little bit of wilderness land. Close to where I live. … my friend…whose dog died six months later. She had ashes from her little dog and I had ashes from another litter rescue that I had for 10–11 years who had passed before. And so they all ran in that little area and it’s not an off leash park, but it was when we went. And they were just all happy there. It was a happy place for all of us.”*

P12. *“I asked for her ashes and I have her paw prints and her ashes are in a very nice container that the vet offered me so she’s sitting on my desk in my suite. My friends, neighbors from upstairs, their daughter painted a picture of her as well and I have another portrait on the wall. So, so she’s, she’s there. You know, so, so it’s good. I’m glad that I have those memories to place around my suite.”*

#### Resuming life again

Most participants stated they began to resume a more active life over time, some without major impactful grief and others even though their major grief continued. For instance, one participant spoke of her enduring loss. P12. *“She (her dog) was there with me through so many things…She was so solid…There was just a real special connection with her…As time has gone on and I’ve had more time, it’s been tough.”*

All reported retaining good memories of their CA, while resuming daily activities and social contact with friends and family. Seven participants also adopted, or fostered, another pet. Several participants spoke about deciding not to start a new CA relationship because of concerns for potentially outliving the CA and worrying about the animal’s welfare if they died, needed to move to a facility that did not allow CAs, or were hospitalized.

P2. *“I think just time, you know, just dealing with time. And then I got the idea of maybe I should be thinking about getting another dog, and I kept putting it off putting it off. And one of my nieces actually said, well, you should, if you want a dog. You should get one.”*

P7. *“It was hard to focus enough on reading, but, you know, doing like even a magazine, buying a decorating magazine and laying the tub to read it, that kind of thing. It didn’t feel like I enjoyed it as much, necessarily. But in retrospect I realize how important it is to build that into your life and not to give that up because you don’t feel like doing it.”*

P6. *“I didn’t sleep very well… But after I started fostering dogs for a (rescue) agency…. And what I realized when I … actually slept better with having the dogs with me.”*

## Discussion

The aim of this multiple-methods study was to develop a greater understanding of the impact of CA death on older women living alone in the community, as well as the supports that should be available to help these individuals with their grief so as to support their personal well-being and thus maintain successful aging-in-place. Of particular note is that while most participants talked about handling their CA grief and regaining personal well-being, their strategies were largely idiosyncratic. What one participant reported as beneficial was not always seen as useful by another. One notable commonality however, was that none of the study participants sought formal help with their grief over losing their CA, such as through pet loss support groups or counseling services. Some participants reported that while they were aware that this was an option, they did not feel comfortable exposing their grief to outsiders. Others who were not aware of these supports similarly felt they would choose not to use them. The interviewed women consequently accepted and managed their grief largely on their own, with some help from a few trusted friends or family members and with various degrees of immediate pre- and post-euthanasia support from a veterinary practice.

Concerns arose when it became evident through this study that they were experiencing disenfranchised grief, as they had their loss minimized or misunderstood by others. Participants had different coping strategies for this, including being very selective over who they told about the CA death. Without social and emotional support, the risk of prolonged or complicated grief exists for older women [1], especially considering the extent of the loss that could be assumed given the high scores found for “Love” on the PALS and for “Grief” on the PBQ. These findings suggest that greater societal knowledge and acceptance of CA-based grief, facilitated emotional support, and socially-endorsed grief rituals, along with improvements in the advertising and perhaps availability of formal bereavement supports, appear necessary to ensure the well-being and successful aging-in-place of older adult women living alone after the loss of a CA. We expand these points below.

### Greater societal knowledge and acceptance of major CA-related grief

This is not the first study that has identified CA death as a significant loss; an individual’s attachment to their pet can be as close, or closer, than for a human companion [5, 9, 26, 27]. In older adulthood, this emotional bond is particularly significant as interpersonal relationships and physical health often decline over time [28–30]. The feelings of shock, disbelief, numbness, anxiety, and depression characteristic of grief after the loss of a close human relationship are mirrored in the experiences of bereaved CA owners [12, 26]. Many of our participants noted a decline in social interactions after the loss of their CA. Loneliness and social isolation have been associated with increased mortality from decreased health and reductions in functional abilities; with these factors compromising an older adult’s independence and potential for successful aging-in-place [18, 19].

CA deaths caused catastrophic (understood as sudden, profound, and highly impactful) grief, among all of the interviewed women. This finding is in keeping with past reports of individuals describing their CA as their child [5, 9]; indeed, some past emotional parallels have been identified between the loss of a pet and experiencing a miscarriage or the loss of a very young child [4, 31]. While our interviews did not reveal this comparison, we did find some similarity as individuals in our study and other studies reported a lack of understanding from other people over the depth of the grief experienced [4, 13, 31]. Moreover, in these situations, it must be recognized that there is often a public expectation of mature women being able to cope with the death and then move on, recovering quickly. This expectation reflects the general lack of recognition of the importance of CAs for older people and the belief that older people will have gained personal abilities over a life time to be able to deal with grief. This breakdown in understanding on the part of others can further alienate and exacerbate the pain of those experiencing CA loss [4]. In the case of bereaved CA owners, the underlying assumption is that the bond between human and CA is not as deep and meaningful as the bond between humans, and so the grief experienced by CA owners may not be recognized as genuine [5, 12, 30]. This aligns with the literature on disenfranchised grief and we propose that it warrants closer attention in designing meaningful interventions for those suffering from CA grief. To avoid prolonged or complicated grief and facilitate positive personal growth after a devastating loss, human or animal, effective coping strategies, social legitimization of grief, and immediate and ongoing social and emotional support are necessary [31].

### Facilitating social and emotional support and grief rituals

Despite the therapeutic effect of memorialization to remember and honor the deceased, there are few socially-acceptable and viable death rituals for people bereaved by CA death [32–34]. If bereaved CA owners are unable to express themselves, the healing process may be complicated or prolonged [5, 12]. Memorialization practices, such as writing a eulogy, can significantly lessen feelings of disenfranchised grief because it provides validation of the relationship and a way to express grief [32, 33]. As reported by a number of our participants, looking at photos or reminiscing over the lost CA can help emotionally sustain the grieving CA owner, despite permanent physical CA separation [32]. While many of the study participants reflected on the positive impact that memorialization practices had on their grief resolution process, these practices were often quite different and ranged from printing and framing photos, making burial plans, cremation ceremonies, and continued conversations with their deceased CA on special occasions. Although not mentioned by our participants, there is some evidence that pastoral care, in the form of prayers or a funeral service for the deceased CA, may be of benefit to some mourners [4]. While social workers, veterinarians, and bereavement counsellors should consider advising the use of memorial devices as necessary coping strategies for vulnerable individuals, [32–34], care must be taken to avoid assumptions about what timing or form these devices should take.

A number of study participants identified changes to their daily routine after the loss of their CA; including the loss of being a caregiver for their CA. Caring for a CA provides individuals with a sense of purpose, while it also maintains a companionship. This work fulfills relationship needs, all of which have a particularly positive effect on the well-being of older adults who may lack access to or involvement in human social networks [35]. As attention to the CA/human bond grows, we see a picture of CAs not only as recipients of care from their human, but also of CAs providing a form of care to their human. That ‘caregiving’ role takes the form of nurturing, and promoting meaningful physical and social activity; key components of successful aging-in-place and well-being. We suggest that the loss of any such ‘CA caregiver’ to an older adult can deepen the magnitude of the loss; similar to the point made about the death of an informal caregiving friend by Stein et al. [36].

After the loss of their CA, some of our study participants chose to adopt a new CA, thus continuing with the benefits previously mentioned, as well as aiding the individual in overcoming their grief through new demands on their activities and energy. While not specifically stated by participants, CAs and the social support that they provide, have been associated with reduced levels of depression and so having a new CA to take care of and to care for them in return may help reduce the risk and combat the negative effects of bereavement [1, 2]. This matter, like the other grief coping strategies shared by participants, was not universal to all participants. Grief intervention choices should not assume CA replacement as being the desired or most effective coping strategy.

### Availability, promotion, and use of bereavement interventions

Despite the potential for extreme long-lasting and impactful grief, it is evident through our study that individuals are largely left on their own to find ways of dealing with the death of their CA. Following a human death, support for the bereaved is commonly offered and public ritualization of grief occurs, however this is not the case after the loss of a CA [5, 7, 9]. The grief associated with the loss of a CA is often ignored or diminished, creating a barrier to healing [5, 7, 9, 13, 30]. Some participants experienced this dismissal of their grief from friends or family, while others chose to tell only select friends or family members of their grief, so as to receive positive support and avoid negative reactions. Dismissive comments from friends, family, or even counsellors regarding the importance of the relationship between human and CA can add to the isolation experienced by bereaved CA owners [4]. This dismissal can cause individuals to suppress their grief, and can create feelings of guilt and shame over the grief they experience [4, 11]. If this grief is not viewed as genuine and individuals are not offered formal support options or compassionate understanding and support from friends and family, typical grief reactions, such as sadness and anger, may be intensified and contribute to complicated grief, an enduring and impactful form of grief [1, 5, 7]. Socially endorsed support during the bereavement period is essential as it guards the mental and physical well-being needed by older adults to maintain independence and quality of life [37].

Although many participants relied on veterinary staff to provide support and confirmation of their decision to euthanize their CA, the skills needed to provide informational and emotional support are often not included in the formal training of veterinary staff. Rather, these skills are learned on the job, through individual experiences [38]. This inconsistent availability of trained staff may impact the CA owner’s experience with euthanasia and then the grief related to the death of their CA. The CA owner’s interaction with veterinarians and veterinarian staff during the euthanasia process has the potential then to relieve or exacerbate their experience of grief related to the CA loss [38, 39]. The literature suggests that counsel from a veterinarian can help ease the guilt often associated with the decision to euthanize. Including formal training for social support in veterinarians and veterinary support staff education can improve the immediate grief support that those bereaved by CA loss receive, helping to reduce the risk of prolonged or complicated grief [38]. Importantly, this role cannot rest solely with veterinary practices, as other healthcare providers and the general public require education or training as well.

Limited public awareness of the significant impact of CA grief, and the disenfranchised nature of CA grief, may prevent individuals from seeking out formal counseling or other grief supports [26]. Awareness and acknowledgement of CA grief by counselors and clinicians can help address the existing gap between the struggles faced by bereaved CA owners and the availability and societal acceptance of CA bereavement supports. When grief related to CA loss is socially normalized and acknowledged as a risk factor for well-being by health and social care professionals, individuals are more likely to seek out and utilize grief supports. Advancements in the availability and acceptance of these supports may also help ease the burden on veterinary professionals who want to ensure their clients’ well-being after the loss of their CA [26].

## Conclusions

This study was an important first step in exploring the experiences of older, community-dwelling, single women over the death of their CA; as well as their knowledge about and use of personal and other resources in dealing with grief and maintaining well-being in their lives. There is limited information on CA grief and recovery in older women, and this study provides insight into this all-too-common phenomenon. The information gathered in this study, although not generalizable to all who experience CA deaths, highlights the importance of acknowledging and addressing CA death and grief to facilitate the well-being and successful aging-in-place of older women, and potentially older men and others in the community. We believe our findings inform future studies for developing and testing resources that accommodate the highly idiosyncratic nature of recovery strategies for CA grief.

## Data Availability

The data set used during the current study is available from the corresponding author on reasonable request.

## Abbreviations

CA: Companion animal
PBQ: Pet Bereavement Questionnaire
PALS: Pet Attachment and Life-Impact Questionnaire
